# Clinical impact of intratumoral HER2 heterogeneity on trastuzumab deruxtecan efficacy in patients with HER2-positive gastric cancer

**DOI:** 10.1007/s10120-026-01736-9

**Published:** 2026-04-02

**Authors:** Koichiro Yoshino, Manabu Takamatsu, Akira Ooki, Keisho Chin, Daisuke Takahari, Hiroki Osumi, Mariko Ogura, Mikako Tamba, Koshiro Fukuda, Keitaro Shimozaki, Shohei Udagawa, Shota Fukuoka, Eiji Shinozaki, Kensei Yamaguchi, Takeru Wakatsuki

**Affiliations:** 1 Department of Gastrointestinal Chemotherapy, The Cancer Institute Hospital of Japanese Foundation for Cancer Research, Tokyo, Japan; 2 Department of Pathology, The Cancer Institute Hospital of Japanese Foundation for Cancer Research, Tokyo, Japan; 3 Division of Pathology, Cancer Institute of Japanese Foundation for Cancer Research, Tokyo, Japan; 4 Division of Medical Oncology, Department of Internal Medicine, Gunma University Graduate School of Medicine, Gunma, Japan; 5 Department of Gastroenterology, Graduate School of Medicine, The University of Tokyo, 7-3-1 Hongo Bunkyo-ku, Tokyo, 113-8655 Japan

**Keywords:** HER2 heterogeneity, Trastuzumab deruxtecan, Gastric cancer, Trastuzumab-free interval, Prognostic biomarker

## Abstract

**Background:**

Intratumoral HER2 heterogeneity has been associated with poor response to trastuzumab-based chemotherapy in HER2-positive gastric cancer. However, its clinical significance in patients treated with trastuzumab deruxtecan (T-DXd) remains unclear.

**Methods:**

Patients with advanced HER2-positive gastric cancer who received T-DXd as second-line or later therapy were enrolled. HER2-positive proportion was defined as the percentage of tumor cells with immunohistochemical score ≥ 2 + in diagnostic specimens obtained before first-line therapy. The cutoff value was determined using time-dependent receiver operating characteristic analysis.

**Results:**

Forty-three patients were enrolled. Median progression-free survival (PFS) and overall survival (OS) were 4.7 and 8.1 months, respectively. With an optimal cutoff of 79%, 20 patients (47%) were in the HER2-homogeneity group and 23 (53%) in the HER2-heterogeneity group. The HER2-homogeneity group showed significantly longer PFS (6.3 vs. 3.1 months, p = 0.002) and OS (9.8 vs. 6.0 months, p = 0.006) with higher overall response rate (79% vs. 19%, p < 0.001). In Cox proportional hazards model, HER2-homogeneity was an independent prognostic factor for both PFS (hazard ratio (HR) 0.37, 95% confidence interval (CI) 0.18–0.76, p = 0.007) and OS (HR 0.41, 95% CI 0.21–0.82, p = 0.01).

**Conclusions:**

Intratumoral HER2 heterogeneity in pre-treatment diagnostic specimens was associated with T-DXd efficacy and may represent a potential prognostic factor.

**Supplementary Information:**

The online version contains supplementary material available at 10.1007/s10120-026-01736-9.

## Introduction

Gastric cancer is the fifth most common malignancy worldwide and the fifth leading cause of cancer-related death, with approximately one million new cases diagnosed annually [1]. Despite advances in surgical techniques and adjuvant therapies, the prognosis for advanced gastric cancer remains poor, with a 5-year overall survival (OS) rates below 20% [2, 3]. The heterogeneous nature of gastric cancer—both histologically and molecularly—contributes substantially to treatment challenges and poor outcomes [4].

Human epidermal growth factor receptor 2 (HER2) is overexpressed in approximately 15–20% of gastric and esophagogastric junction (EGJ) cancers, and serves as an important therapeutic target [5, 6]. The pivotal Trastuzumab for Gastric Cancer (ToGA) trial established trastuzumab combined with fluoropyrimidine and platinum as the standard first-line regimen for HER2-positive advanced gastric cancer, demonstrating significant survival benefits compared with chemotherapy alone [7]. Recent clinical trials have further refined the treatment landscape for HER2-positive gastric cancer. The KEYNOTE-811 trial established pembrolizumab plus trastuzumab and chemotherapy as the standard first-line therapy for patients with PD-L1 CPS ≥ 1 [8]. Furthermore, the HERIZON-GEA-01 trial demonstrated that zanidatamab, a bispecific HER2-directed antibody targeting HER2 extracellular domains 2 and 4, in combination with chemotherapy, with or without tislelizumab (an anti–PD-1 antibody), improved progression-free survival (PFS) compared with trastuzumab-based chemotherapy in the first-line setting, while the triplet regimen also demonstrated a significant OS benefit, further expanding HER2-targeted treatment options [9].

Several candidate biomarkers for trastuzumab-based chemotherapy have been explored [10–15]. Among them, the clinical significance of intratumoral HER2 heterogeneity in gastric cancer has emerged as an important consideration. HER2 heterogeneity, characterized by variable HER2 expression levels within the same tumor, ranges in frequency from 50–75.4% [13, 14, 16] and has been associated with poor outcomes to trastuzumab-based therapy in HER2-positive gastric cancer [13, 14]. Notably, loss of HER2 expression after first-line trastuzumab-based therapy has been reported in 25–69% of patients [17–21], raising concerns about the efficacy of subsequent anti-HER2 therapies.

Trastuzumab deruxtecan (T-DXd) is a novel HER2-targeted antibody–drug conjugate with notable efficacy in HER2-positive gastric cancer [22]. T-DXd consists of an anti-HER2 antibody linked via a cleavable linker to a topoisomerase I inhibitor payload enabling targeted delivery of cytotoxic agents to HER2-expressing cancer cells [23]. The unique mechanism of action of T-DXd, including its high drug-to-antibody ratio and membrane-permeable payload with a bystander killing effect may help overcome some limitations posed by HER2 heterogeneity that affect conventional anti-HER2 therapies [24, 25]. The DESTINY-Gastric01 trial demonstrated superior OS with T-DXd compared with physician’s choice chemotherapy in patients previously treated with trastuzumab-based regimens, without requiring re-biopsy for HER2 re-evaluation [26]. More recently, the DESTINY-Gastric04 trial demonstrated the efficacy of T-DXd monotherapy when HER2 positivity is confirmed immediately before treatment [27]. In this evolving therapeutic context, identifying biomarkers that predict treatment response to T-DXd has become increasingly important.

Given these considerations, whether intratumoral HER2 heterogeneity in diagnostic specimens obtained prior to first-line therapy predicts clinical outcomes of T-DXd remains an important question awaiting elucidation. This study aims to evaluate the clinical significance of HER2 heterogeneity on the efficacy and safety of T-DXd in patients with HER2-positive gastric and EGJ cancers.

## Methods

### Study design

This retrospective, single-institution study was conducted at the Cancer Institute Hospital of the Japanese Foundation for Cancer Research (JFCR). Eligible patients were aged ≥ 18 years, with histologically confirmed unresectable, advanced, or recurrent gastric or EGJ cancer and an Eastern Cooperative Oncology Group (ECOG) performance status (PS) of 0–2. Patients received T-DXd as second-line or later therapy between May 2018 and August 2023. Availability of glass slides for HER2 immunohistochemistry (IHC) before first-line treatment were required. The study was approved by the Institutional Review Board of the JFCR (registration number: 2015–1029) and conducted in accordance with the Declaration of Helsinki. Written informed consent was waived owing to the opt-out procedure described on the hospital website.

### HER2 and CLDN18.2 assessment

HER2 expression was evaluated using surgical specimens in patients who underwent tumor resection and biopsy specimens in patients with unresectable disease. In our institution, the standard protocol for gastric cancer biopsies at this facility requires obtaining at least five biopsy specimens from the tumor whenever possible [28]. Cases with fewer than two specimens containing tumors were excluded. For surgical specimens, a single representative tumor section was selected by a pathologist based on H&E staining for HER2 assessment, in accordance with the Japanese Society of Pathology guidelines.

HER2 status was assessed using immunohistochemistry (IHC) and in situ hybridization (ISH) according to the recommendations for gastric cancer. IHC was performed using the HercepTest™ (Dako / Agilent Technologies, Santa Clara, CA, USA), a companion diagnostic test for trastuzumab. Tumors were scored as IHC 0, 1 + , 2 + , or 3 + based on the intensity and pattern of membranous staining. For cases with IHC 2 + , ISH was performed using the HER2 IQFISH pharmDx™ Kit (Dako / Agilent Technologies) to determine HER2 gene amplification status. Tumors were considered HER2-positive if they showed IHC 3 + or IHC 2 + with ISH-positive results (HER2 / CEP17 ratio ≥ 2.0).

The HER2-positive proportion was defined as the percentage of tumor cells with an IHC score ≥ 2 + among all tumor cells in pre-treatment diagnostic specimens. The HER2-positive proportion was evaluated blinded to clinical information.

CLDN18.2 expression was evaluated using the VENTANA CLDN18 (43-14A) RxDx Assay (Roche Diagnostics, Basel, Switzerland), a companion diagnostic test for zolbetuximab. This assay detects the CLDN18 protein; however, as gastric cancer exclusively expresses the CLDN18.2 isoform, staining results were interpreted as CLDN18.2 expression. Tumors with ≥ 75% moderate-to-strong membranous staining were classified as CLDN18.2-positive. The percentage of tumor cells with moderate-to-strong (≥ 2 +) membranous staining was assessed by the pathologist at our institution.

The cutoff value for the HER2-positive proportion was determined using time-dependent receiver operating characteristic (ROC) analysis, with prediction time set to the median OS. Tumors with a HER2-positive proportion equal to or above the cutoff value were classified into the HER2*-*homogeneity group, and those below the cutoff into the HER2-heterogeneity group. For patients who underwent re-biopsy before T-DXd initiation, we examined the changes in HER2 expression relative to before first-line treatment.

### Treatment and clinical assessment

Patients received T-DXd at 6.4 mg/kg every 3 weeks, with dose reductions as needed for adverse events (AEs). Treatment was continued until disease progression, unacceptable AEs, or patient refusal. Tumor assessments were conducted using computed tomography (CT). Treatment response was evaluated according to the Response Evaluation Criteria in Solid Tumors (RECIST) version 1.1 in patients with at least one measurable lesion. Objective response rate (ORR) was defined as the proportion of patients with complete response (CR) or partial response (PR). Disease control rate (DCR) refers to the proportion of patients achieving CR, PR, or stable disease (SD). Patient characteristics, AE, and clinical outcomes were extracted from electronic medical records. Clinicopathological data included age, sex, ECOG PS primary site, metastatic sites, number of metastatic organs, prior treatment, and trastuzumab-free interval (TFI). Hematological and non-hematological toxicities were assessed using the National Cancer Institute Common Terminology Criteria for Adverse Events (version 5.0).

### Statistical analysis

Associations between the HER2-positive proportion and OS, PFS, ORR, and safety were analyzed. Median follow-up was defined as the time from treatment initiation to the last confirmed survival for cases with censored data. OS was measured from treatment initiation to death or last confirmation of survival, and PFS from treatment initiation to disease progression or death. TFI was defined as the interval between the last trastuzumab administration and the first T-DXd administration. The cutoff for TFI was determined by time-dependent ROC analysis using median survival time of the population as the reference.

Survival curves were estimated using the Kaplan–Meier method, and correlations were tested using the log-rank test. Statistical significance was set at two-sided p < 0.05. Cox proportional hazards analysis was performed by selecting variables with p < 0.05 in the univariate analysis, and including them in multivariate model, thereby providing adjusted hazard ratios (HRs) with 95% confidence intervals (CIs). Categorical variables were summarized as frequencies and proportions, and continuous variables as medians with ranges. Fisher’s exact test was used for categorical comparisons, and the Mann–Whitney U test was used for continuous variables. Correlations between continuous variables were assessed using Spearman’s correlation coefficient with a two-tailed *t*-test. All analyses were performed using EZR version 1.55 (Saitama Medical Center, Jichi Medical University), a graphical interface for R and R Commander [29].

## Results

### Patient characteristics

The baseline characteristics of the 43 patients are summarized in Table 1. The median age was 67 (range, 31–82) years, and 70% were male. ECOG PS was 0 in 15 patients (35%), 1 in 24 patients (56%), and 2 in 4 patients (9%). Eight patients (19%) had tumors located in the EGJ, and 12 (28%) had recurrent gastric cancer after surgery. T-DXd was administered as third-line therapy in 28 patients (65%), and as fourth-line or later therapy in 14 patients (33%). Metastases involved two or more organs in 37 patients (86%), with lymph node metastases in 29 (67%), peritoneal dissemination in 26 (60%), and liver metastases in 22 (51%). HER2 IHC 2 + / FISH-positive tumors were observed in 10 patients (23%).

**Table 1:** Patient characteristics

Characteristics	No. of total patients (%) n = 43	HER2-homo group (%) n = 20	HER2-hetero group (%) n = 23	p value
Age				0.97
Median [range], years	67 [31–82]	68 [31–82]	68 [40–79]	
Sex				1.00
Male	30 (70)	14 (70)	16 (70)	
Female	13 (30)	6 (30)	7 (30)	
ECOG PS				0.12
0	15 (35)	10 (50)	5 (22)	
1	24 (56)	8 (40)	16 (70)	
2	4 (9)	2 (10)	2 (8)	
Primary site				1.00
Esophago-gastric junction	8 (19)	4 (20)	4 (17)	
Stomach	35 (81)	16 (80)	19 (83)	
Disease status				1.00
Unresectable	31 (72)	15 (75)	16 (70)	
Reccurent	12 (28)	5 (25)	7 (30)	
Lauren’s classification				0.54
Diffuse type	18 (42)	7 (35)	11 (48)	
Intestinal type	25 (58)	13 (65)	12 (52)	
Treatment line				0.74
2	1 (2)	1 (5)	0 (0)	
3	28 (65)	13 (65)	15 (65)	
≥ 4	14 (33)	6 (30)	8 (35)	
No. of metastatic organ				0.10
1	6 (14)	1 (5)	5 (22)	
2	19 (44)	12 (60)	7 (30)	
≥ 3	18 (42)	7 (35)	11 (48)	
Metastatic Organ				
Lymph nodes	29 (67)	15 (75)	14 (61)	0.35
Peritoneal dissemination	26 (60)	8 (40)	18 (78)	0.01
Liver	22 (51)	13 (65)	9 (39)	0.13
HER2 status				< 0.001
IHC 2 + / FISH +	10 (23)	0 (0)	10 (43)	
IHC 3 +	33 (77)	20 (100)	13 (57)	
Trastuzumab-free Intetrval				
Median [range], months	7.6 [1.6–33.6]	9.2 [1.6–33.6]	5.8 [2.1–32.7]	0.55
Sample				1.00
biopsy specimen	34 (79)	16 (80)	18 (78)	
surgical specimen	9 (21)	4 (20)	5 (22)	
Biopsy specimens, median [range]				
total pieces	6 [2–13]	7 [2-9]	6 [3-13]	0.38
tumor-containing pieces	4.5 [2–8]	5 [2–8]	4 [2–8]	0.13
HER2-positive proportion				
Median [range], %	63 [0–100]	95 [79–100]	35 [0–68]	< 0.001

Abbreviations ECOG, eastern cooperative oncology group, HER2, human epidermal growth factor receptor 2, FISH,fluorescence in situ hybridization, homo, homogeneity, hetero, heterogeneity, IHC, immunohistochemistry, PS,performance status

### HER2 heterogeneity analysis

HER2 expression was evaluated using biopsy specimens in 34 patients (79%) and surgical specimens in 9 patients (21%). In this cohort, the median number of biopsy specimens obtained was 6 (range: 2–13), with a median of 4.5 specimens (range: 2–8) containing sufficient tumor tissue for evaluation. HER2 heterogeneity was assessed by evaluating all available tumor-containing specimens from each patient.

Among the 34 biopsy-assessed patients, the number of biopsy specimens (median: 7 (range 2–9) vs. 6 (range 3–13), p = 0.38) and tumor-containing specimens (median: 5 (range 2–8) vs. 4 (range 2–8), p = 0.13) did not differ significantly between the HER2-homogeneity and HER2-heterogeneity groups. The proportion of surgical-based HER2 assessments was also comparable between the two groups (4 of 20 [20%] vs. 5 of 23 [22%], p = 1.00) (Table 1).

**Fig. 1:**
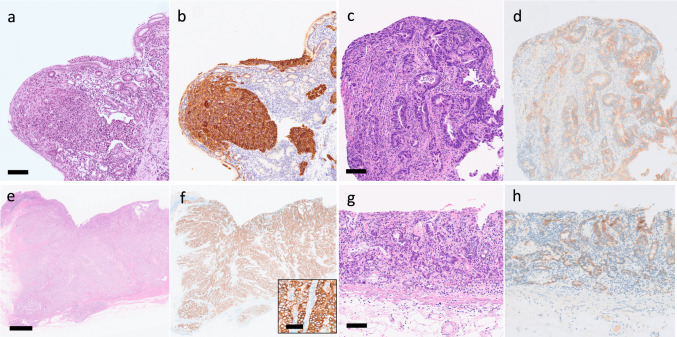
Immunohistochemistry of HER2 for gastric cancer specimens. A biopsy specimen showing 100% positivity in (**b**), while 52% in (**d**). A surgically resected specimen showing 100% in (**f**), while 40% in (**h**). Scale bars: 100 µm in (**a**), (**c**), (**f**) (insert) and (**g**), and 2 mm in (e). Abbreviations: HER2, human epidermal growth factor receptor 2

A time-dependent ROC curve identified an optimal HER2-positive proportion cutoff of 79% for stratifying OS (Figure S1a). Tumors with ≥ 79% HER2 cells were classified as the HER2-homogeneity group, and those with < 79% as the HER2-heterogeneity group (Fig. 1); of the 43 patients, 20 (47%) and 23 (53%) were classified into these groups, respectively. Compared with the HER2-homogeneity group, the HER2-heterogeneity group had significantly more patients with peritoneal dissemination (8 (40%) vs. 18 (78%), p = 0.01), HER2 IHC score 2 + tumors (0 (0%) vs. 10 (43%), p < 0.001), and lower HER2-positive proportion (95% vs. 63%, p < 0.001) (Table 1). Median TFI was 7.6 months (range 1.6–33.6), which was slightly longer in the HER2-homogeneity group (9.2 months, range 1.6–33.6) than in the HER2-heterogeneity group (5.8 months, range 2.1–32.7); however, no significant difference was observed between the two groups (p = 0.55).

### CLDN18.2 expression

CLDN18.2 expression was evaluated in 42 of 43 patients; one patient in the HER2-heterogeneous group could not be assessed due to insufficient tumor tissue. The median percentage of tumor cells with moderate-to-strong (≥ 2 +) CLDN18.2 membranous staining was 35% (range: 0–100%) in the HER2-homogeneity group and 65% (range: 0–100%) in the HER2-heterogeneity group (p = 0.16). (Table 2, Figure S6a).

**Table 2:** CLDN18.2 expression

CLDN18.2 expression	No. of total patients (%) n = 42	HER2-homo group (%) n = 20	HER2-hetero group (%) n = 22*	p value
CLDN18.2 positive tumor cell				
Median [range], %	50 [0–100]	35 [0–100]	65 [0–100]	0.16
CLDN18.2 expression level				0.33
0%	6 (14)	5 (25)	1 (5)	
1–49%	14 (33)	6 (30)	8 (36)	
50–74%	3 (7)	1 (5)	2 (9)	
≥ 75%	19 (45)	8 (40)	11 (50)	
CLDN18.2 status				0.55
Positive	19 (45)	8 (40)	11 (50)	
Negative	23 (55)	12 (60)	11 (50)	

^*^ CLDN18.2 data could not be obtained from one patient in the HER2-hetero group

Abbreviations HER2, human epidermal growth factor receptor 2, homo, homogeneity, hetero, heterogeneity

CLDN18.2 was positive in 45% (19 / 42) of all cases, specifically 40% (8 / 20) of the HER2-homogeneity group and 50% (11 / 22) of the HER2-heterogeneity group. Among patients with IHC 2 + / ISH + tumors (all HER2-heterogeneous), 50% (5 / 10) showed CLDN18.2 positivity. Among patients with IHC 3 + tumors, CLDN18.2 positivity was observed in 50% (6 / 12) of HER2-heterogeneous cases (p = 0.52).

### Efficacy outcomes

**Fig. 2:**
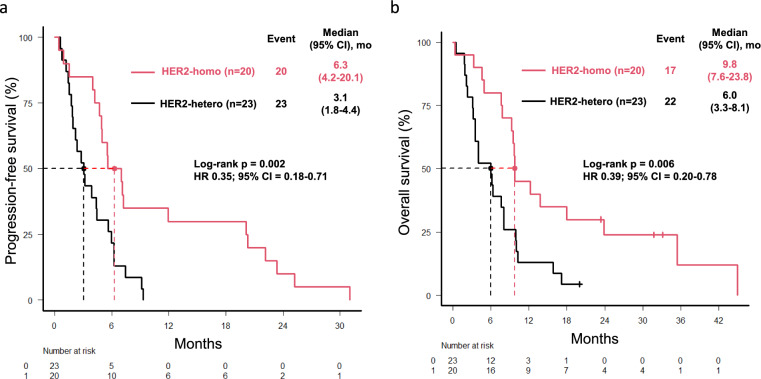
Kaplan–Meier estimates of PFS (**a**) and OS (**b**). The HER2-homogeneity group was defined as having a HER2-positive proportion of 79% or higher, while the HER2-heterogeneity group was defined as having a HER2-positive proportion of less than 79%. Abbreviations: PFS, progression-free survival; OS, overall survival; CI, confidence interval

At a median follow-up of 24.5 months, the median PFS was 4.7 months (95% CI, 3.1–6.0; Figure S2a), and the median OS was 8.1 months (95% CI, 4.9–9.8; Figure S2b). Among 30 patients with measurable lesions, ORR and DCR were 47% and 73%, respectively (Table 3). Median PFS (mPFS) was significantly longer in the HER2-homogeneity group than in the HER2-heterogeneity group (6.3 months, 95% CI, 4.2–20.1 vs. 3.1 months, 95% CI, 1.8–4.4, p = 0.002; Fig. 2a). Similarly, the median OS (mOS) was longer in the HER2-homogeneity group than in the HER2-heterogeneity group (9.8 months, 95% CI, 7.6–23.8 vs. 6.0 months, 95% CI, 3.3–8.1, p = 0.006; Fig. 2b). In univariate analysis, HER2-homogeneity was significantly associated with longer PFS (HR 0.35, 95%CI 0.18–0.71, p = 0.004) and OS (HR 0.39, 95% CI, 0.20–0.78, p = 0.008) (Table 4). In multivariable Cox proportional hazards analysis, HER2-homogeneity was a favorable independent prognostic factor for both PFS (HR 0.37, 95% CI, 0.18–0.76, p = 0.007) and OS (HR 0.41, 95% CI, 0.21–0.82, p = 0.01) (Table 4). ORR was significantly higher in the HER2-homogeneity group (ORR 79% vs. 19%; p < 0.001) (Table 3).

**Table 3:** Best response

Best response	No. of total patients (%) n = 30*	HER2-homo group (%) n = 14	HER2-hetero group (%) n = 16	p value
Complete response	0 (0)	0	0	
Partial response	14 (47)	11 (79)	3 (19)	
Stable disease	8 (27)	2 (14)	6 (38)	
Progressive disease	7 (23)	1 (7)	6 (38)	
Not evaluable	1 (3)	0	1 (6)	
Overall response rate	47%	79%	19%	< 0.001
Disease control rate	73%	93%	56%	0.13

^*^Cases with target lesions

Abbreviations HER2, human epidermal growth factor receptor 2, homo, homogeneity, hetero, heterogeneity

**Table 4:** Cox proportional hazards analysis

Factors	N	Univariate (PFS)			Multivariate (PFS)			Univariate (OS)		Multivariate (OS)	
		mPFS [95% CI]	HR [95% CI]	p	HR [95% CI]	p	mOS [95% CI]	HR [95% CI]	p	HR [95% CI]	p
Gender											
Female	13	6.0 [1.8–7.0]	Ref				9.0 [4.6–12.2]	Ref			
Male	30	4.4 [2.8–5.6]	1.01 [0.52–1.96]	0.99			8.0 [4.0–9.8]	1.14 [0.56–2.31]	0.72		
Age											
< 65	18	3.7 [1.9–6.0]	Ref				7.9 [4.1–10.0]	Ref			
≥ 65	25	5.5 [3.9–7.1]	0.90 [0.49–1.67]	0.74			8.1 [4.0–10.3]	1.06 [0.55–2.04]	0.86		
ECOG PS											
0, 1	39	4.9 [3.2–6.2]	Ref		Ref		8.1 [4.9–10.1]	Ref			
2	4	2.1 [0.4–NA]	3.36 [1.14–9.88]	0.03	3.42 [1.13–10.4]	0.03	5.2 [0.4–NA]	2.60 [0.89–7.61]	0.08		
Primary location											
Stomach	35	5.0 [3.1–6.2]	Ref				8.1 [6.0–10.0]	Ref			
Esophago-gastric junction	8	4.1 [0.7–11.9]	0.91 [0.40–2.07]	0.82			5.8 [2.2–18.0]	1.09 [0.47–2.50]	0.84		
Lauren classification											
Intestinal type	25	5.0 [2.8–7.2]	Ref				8.1 [4.0–10.3]	Ref			
Diffuse type	18	4.4 [1.9–6.2]	1.45 [0.77–2.73]	0.25			8.6 [4.1–10.1]	1.07 [0.56–2.06]	0.84		
No. of metastatic organs											
1, 2	25	5.0 [3.2–7.2]	Ref				9.8 [4.9–15.9]	Ref			
≥ 3	18	4.3 [1.5–6.0]	1.59 [0.85–2.97]	0.15			6.3 [3.3–7.9]	1.93 [1.00–3.73]	0.051		
Liver metastasis											
Absent	21	3.2 [1.5–6.2]	Ref				8.1 [3.3–12.2]	Ref			
Present	22	5.3 [3.9–7.1]	0.73 [0.40–1.36]	0.32			8.0 [4.9–9.8]	0.99 [0.52–1.90]	0.98		
Peritoneal dissemination											
Absent	17	5.6 [2.3–9.3]	Ref				9.7 [4.0–18.0]	Ref			
Present	26	4.1 [1.9–6.0]	1.75 [0.91–3.38]	0.09			7.1 [3.6–10.0]	1.57 [0.80–3.09]	0.19		
No. of previous chemotherapy											
≤ 3	29	4.4 [2.3–7.0]	Ref				7.7 [4.1–10.0]	Ref			
≥ 4	14	5.3 [1.2–6.2]	1.06 [0.56–2.03]	0.85			9.3 [3.3–13.8]	1.11 [0.57–2.15]	0.76		
Disease status											
Unresectable	31	4.4 [2.8–5.5]	Ref				7.9 [4.6–9.8]	Ref			
Reccurent	12	6.1 [1.2–11.9]	0.80 [0.40–1.58]	0.52			9.9 [2.4–NA]	0.66 [0.31–1.40]	0.28		
Trastuzumab-free interval											
< 5.8 months	19	2.8 [1.5–6.0]	Ref		Ref		4.6 [3.2–8.1]	Ref		Ref	
≥ 5.8 months	24	5.3 [4.2–9.1]	0.51 [0.27–0.97]	0.04	0.60 [0.31–1.15]	0.12	9.8 [7.6–17.2]	0.44 [0.22–0.85]	0.01	0.46 [0.23–0.89]	0.02
HER2 status											
IHC 2 + / FISH +	10	3.1 [0.6–6.2]	Ref				3.8 [0.6–6.4]	Ref			
IHC 3 +	33	4.9 [3.2–6.2]	0.65 [0.31–1.34]	0.24			9.5 [7.6–10.1]	0.51 [0.24–1.09]	0.08		
CLDN18.2 status											
Negative	23	6.2 [4.0–7.5]	Ref				9.6 [6.2–13.8]	Ref			
Positive	19	3.2 [1.5–4.7]	1.85 [0.98–3.49]	0.06			6.0 [3.3–9.5]	1.81 [0.94–3.5]	0.08		
HER2-heterogeneity											
Heterogeneity	23	3.1 [1.8–4.4]	Ref		Ref		6.0 [3.3–8.1]	Ref		Ref	
Homogeneity	20	6.3 [4.2–20.1]	0.35 [0.18–0.71]	0.004	0.37 [0.18–0.76]	0.007	9.8 [7.6–23.8]	0.39 [0.20–0.78]	0.008	0.41 [0.21–0.82]	0.01

Abbreviations CI, confidential interval, ECOG, Eastern Cooperative Oncology Group, FISH, fluorescence in situ hybridization, HER2, human epidermal growth factor receptor 2, hetero, heterogeneity, homo, homogeneity, HR, hazard ratio, IHC, immunohistochemitstry, mPFS. median progression-free survival, mOS, median overall survival, NA, not applicable, No, number, PS, performance status, Ref, refference

When stratified by HER2 IHC scoring, IHC 3 + tumors (n = 33) showed mPFS of 4.9 months (95% CI 3.2–6.2) and mOS of 9.5 months (95% CI 7.6–10.1), while IHC 2 + / ISH-positive tumors (n = 10) showed mPFS of 3.1 months (95% CI 0.6–6.2) and mOS of 3.8 months (95% CI 0.6–6.4). (Table 4). Among patients with evaluable best response, IHC 3 + tumors (n = 24) showed an ORR of 50% and DCR of 79%, while IHC 2 + / ISH-positive tumors (n = 6) showed an ORR of 33% and DCR of 50% (Table S1a).

Among the 33 patients with IHC 3 + tumors, when further stratified by HER2 heterogeneity, the HER2-homogeneity group (n = 20) showed mPFS of 6.3 months and the HER2-heterogeneity group (n = 13) showed mPFS of 3.1 months (p = 0.003). Among the 24 IHC 3 + patients with evaluable best response, the HER2-homogeneity group (n = 14) showed an ORR of 79% and DCR of 93%, while the HER2-heterogeneity group (n = 10) showed an ORR of 10% and DCR of 60% (p = 0.003 for ORR) (Table S1b).

### Prognostic value of trastuzumab-free interval

Similarly to the HER2-positive proportion, the optimal cutoff point for TFI was calculated to be 5.8 months using time-dependent ROC analysis (Figure S1b). The TFI-long group was defined as those with a TFI of 5.8 months or longer, and the TFI-short group as those with a TFI of less than 5.8 months.

**Fig. 3:**
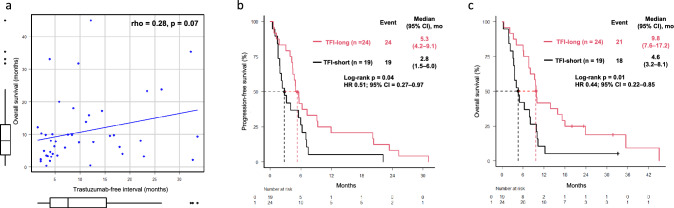
Scatter plot and regression line (**a**), Kaplan–Meier estimates of PFS (**b**) and OS (**c**). Spearman’s rank correlation coefficient was 0.28. The TFI-long group was defined as those with a TFI of 5.8 months or longer, and the TFI-short group as those with a TFI of less than 5.8 months. Abbreviations: PFS, progression-free survival; OS, overall survival; CI, confidence interval; HER2, human epidermal growth factor receptor 2; OS, overall survival; TFI, trastuzumab free interval

TFI and OS showed a weak correlation (rho = 0.28, p = 0.07; Fig. 3a), and the TFI-long group had significantly longer PFS than the TFI-short group. (5.3 vs. 2.8 months, p = 0.04; Fig. 3b). Furthermore, a significant prolongation of OS was observed (9.8 vs. 4.6 months, p = 0.01; Fig. 3c). In multivariable analysis, TFI emerged as an independent prognostic factor for OS (HR 0.46, 95% CI, 0.23–0.89, p = 0.02; Table 4). ORR tended to be higher in the TFI-long group compared with the TFI-short group (61% vs. 25%, p = 0.07; Table S2a).

TFI showed a moderate correlation with OS in the HER2-homogeneity group (rho = 0.41, p = 0.07; Figure S3a). The TFI-long group had significantly longer PFS (9.6 vs. 4.8 months, p = 0.05; Figure S3b) than the TFI-short group. The TFI-long group was also associated with a longer OS (15.9 vs. 6.4 months, p = 0.06; Figure S3c), although there was no statistically significant difference between the TFI-long group and the TFI-short group. On the other hand, no correlation with TFI and OS was observed in the HER2-heterogeneity group (rho = 0.14, p = 0.54; Figure S4a). No significant difference in PFS or OS was observed between the TFI-long group and the TFI-short group (PFS 4.1 vs. 2.3 months, p = 0.41; Figure S4b. OS 7.0 vs. 3.6 months, p = 0.16; Figure S4c).

### Dynamics of HER2-positive proportion

We evaluated the association between dynamic HER2-positive proportion changes after treatment and clinical outcomes. Of the 43 cases in which HER2-positive proportion was assessed at diagnosis, 8 patients underwent re-biopsy prior to administration of T-DXd. Among these, HER2 status turned negative in 2 patients (25%): one had been diagnosed as IHC2 + before treatment, and the other as IHC3 + , but the HER2-positive proportion was 20%. HER2-positive proportion increased in 5 patients (62.5%), remained unchanged in 1 patient (12.5%), and decreased in 2 patients (25%) (Figure S5a).

Using the 79% cutoff in re-biopsy specimens, OS was significantly longer in patients with high HER2-positive proportion (17.0 vs. 2.7 months, p = 0.03; Figure S5c). On the other hand, PFS tended to be longer in the HER2-homogeneity group, although not statistically significant (13.2 vs. 2.1 months, p = 0.17; Figure S5b). The ORR did not differ significantly between the two groups (Table S2b).

### Safety

Grade ≥ 3 treatment-related AEs are summarized in Table 5. Interstitial lung disease (ILD) occurred in 9 patients (21%), all Grade 1, and tended to be more frequent in the HER2-homogeneity group, although the difference was not significant (35% vs. 9%, p = 0.06). Conversely, anorexia was more frequent in the heterogeneity group than in the homogeneity group (0% vs. 17%, p = 0.11).

**Table 5:** Grade 3 or higher adverse events

Adverse events	No. of total patients (%) n = 43	HER2-homo group (%) n = 20	HER2-hetero group (%) n = 23	p value
Neutropenia	7 (16)	4 (20)	3 (13)	0.69
Anorexia	4 (9)	0	4 (17)	0.11
Fatigue	4 (9)	1 (5)	3 (13)	0.61
Thrombocytopenia	1 (2)	0	1 (4)	1.00
Diarrea	1 (2)	0	1 (4)	1.00
Febrile neutropenia	1 (2)	0	1 (4)	1.00
Total	15 (35)	5 (25)	10 (43)	0.34
Interstital lung disease*	9 (21)	7 (35)	2 (9)	0.06

^*^ All cases were Grade 1

Abbreviations HER2, human epidermal growth factor receptor 2, homo, homogeneity, hetero, heterogeneity

### Treatment discontinuation and subsequent therapy

Reasons for treatment discontinuation included PD, AE, and poor PS (Table S3a), with no significant differences between HER2-positive proportion groups. Subsequent therapy was received by 65% and 48% of patients in the HER2-homogeneity and HER2-heterogeneity groups, respectively; however, the difference was not significant (Table S3b).

## Discussion

This study identified several key findings. First, intratumoral HER2 heterogeneity in pre-treatment diagnostic specimens was associated with T-DXd efficacy in patients with HER2-positive gastric cancer, although previous studies have reported frequent HER2 loss after first-line therapy. Second, when limited to patients with IHC 3 + tumors exhibiting HER2- homogeneity, the ORR increased to 79%. Third, longer TFI was significantly associated with prolonged OS, especially TFI showed a moderate correlation with OS in the HER2-homogeneity group. Finally, a higher incidence of ILD was observed in the HER2-homogeneity group. These results suggest that intratumoral HER2 heterogeneity in pre-treatment specimens may represent a potential prognostic factor.

The DESTINY-Gastric01 trial reported an mPFS of 5.6 months and mOS of 12.5 months, with ORR of 51% [23]. Real-world Japanese data reported mPFS of 3.9–4.6 months, mOS of 6.5–8.9 months, and ORR of 41.0–42.9% [30, 31]. In this study, PFS, OS, and ORR were lower than DESTINY-Gastric01 but comparable to real-world outcomes (mPFS 4.7 months, mOS 8.1 months, ORR 47%), likely reflecting inclusion of patients typically excluded from clinical trials due to comorbidities or PS.

The most significant finding is that pre-treatment intratumoral HER2 heterogeneity was associated with T-DXd efficacy, despite frequent HER2 loss after first-line therapy—a known mechanism of resistance to second-line anti-HER2 therapies. HER2 loss occurs in 25–69% of patients following first-line trastuzumab-based therapy [17–21]. Previous pivotal trials showed limited benefit of continuing anti-HER2 therapy beyond first-line treatment. The TyTAN trial found no improvement with lapatinib plus paclitaxel versus paclitaxel alone [32], the GATSBY trial failed to demonstrate T-DM1 superiority over taxane monotherapy (docetaxel or paclitaxel) [33], and the T-ACT study showed that trastuzumab beyond progression did not improve efficacy [18]. The DESTINY-Gastric04 trial, therefore, requires re-biopsy before T-DXd administration to ensure HER2 positivity, and demonstrated its superiority over ramucirumab plus paclitaxel in the second-line treatment setting [27]. However, re-biopsy procedures pose significant challenges, including patient burden, technical difficulties obtaining adequate tissue, potential treatment delays, and high medical costs, highlighting the need for a minimally invasive, practical biomarker. Our finding suggests that assessing HER2 heterogeneity in pre-treatment specimens could represent a useful biomarker for predicting efficacy of T-DXd use without routine re-biopsy. Nevertheless, this does not apply to the use of T-DXd in the second-line setting, and in accordance with the DESTINY-Gastric04 trial, a biopsy should be performed to reassess and confirm the HER2 status. For third-line or later therapy, routine re-biopsy is not recommended in the Japanese Gastric Cancer Association guidelines, HER2 heterogeneity assessment from prior specimens may provide prognostic information.

Several factors may explain why pre-treatment HER2 heterogeneity remains predictive despite HER2 loss. First, HER2 diagnostic variability reflects tumor heterogeneity itself. Repeated HER2 assessment on the same tumor block shows a 22.7% rate of discordance [34]. Studies using matched biopsy and resection specimens reported that the false-negative rate of endoscopic biopsy ranges from 14.3–28.4% [35, 36]. Therefore, some of the observed HER2 loss may merely reflect HER2 heterogeneity rather than true HER2 loss. Haffner et al. reported that patients with discordant HER2 assessments had significantly worse prognosis than those with consistently positive HER2 results [34]. Similarly, Maron et al. performed multiple HER2 assessments and reported that patients with at least one IHC score ≤ 2 + had significantly worse prognosis compared with those who consistently had IHC 3 + results [37]. Crucially, this diagnostic instability manifests intratumoral HER2 heterogeneity, which predicts treatment resistance.

Second, HER2 loss propensity relates to initial HER2 expression levels. The GASTHER3 study revealed that tumors with high HER2 expression are less likely to lose HER2 positivity, while those with lower expression are more prone to HER2 loss [19]. In the current study, among eight patients who underwent re-biopsy, two showed HER2 loss: one was IHC 2 + before treatment, and the other, although IHC 3 + , had only 20% HER2-positive cells, which is consistent with previous data. This suggests tumors with homogeneous, high-level HER2 expression tend to maintain their HER2-positive phenotype throughout treatment, preserving susceptibility to HER2-targeted therapies, whereas it is likely that tumors with heterogeneous, low-level HER2 expression are prone to HER2 loss and consequently show reduced efficacy of second HER2-targeted therapy.

Third, HER2-positive gastric cancer cells may reemerge during periods without anti-HER2 therapy. Some studies performing serial circulating tumor DNA (ctDNA) analysis have reported dynamic changes in *ERBB2* amplification; *ERBB2* copy numbers typically decrease in responders but increase upon disease progression during anti-HER2 therapy [38–43]. In addition, previous studies have suggested an association between longer TFI and favorable survival with T-DXd [30, 31], and this finding was also observed in the current study, specifically in the HER2-homogeneity group, showing particularly favorable outcomes (PFS, 9.6 months; OS, 15.9 months). These data suggest that in certain tumors exhibiting HER2 homogeneity, HER2-positive gastric cancer cells may re-emerge after the release of selection pressure from trastuzumab, similar to the phenomenon observed with anti-EGFR antibodies in colorectal cancer [44]. In summary, HER2 loss appears to occur mainly in tumors exhibiting HER2 heterogeneity, whereas tumors with HER2 homogeneity may maintain HER2 expression during trastuzumab treatment or re-emerge during TFI, indicating that the characteristics of HER2-positive gastric cancer present before first-line therapy may be largely preserved.

The molecular basis for heterogeneity-associated treatment resistance has been further elucidated by recent comprehensive biomarker analyses from the DESTINY-Gastric01 trial [45]. This pivotal study demonstrated that patients with IHC 2 + / ISH + tumors, which frequently exhibit heterogeneous HER2 expression patterns, showed significantly lower ORR than IHC 3 + cases (28.6% vs. 58.2%, respectively). More importantly, patients with high HER2 mRNA expression had markedly higher response rates compared to those with low expression (81.2% vs. 23.5%). The study also revealed that patients with plasma HER2 amplification in ctDNA achieved superior response rates compared to those without amplification (60.6% vs. 34.2%). These molecular findings strongly correlate with histologic observations of HER2 heterogeneity, suggesting that heterogeneous tumors harbor inherently distinct biological features (e.g., lower HER2 mRNA expression and reduced circulating HER2 amplification) that fundamentally limit response to HER2-targeted therapies, including agents with novel mechanisms, such as T-DXd [34, 46].

Our HER2 heterogeneity assessment successfully enriched for exceptional responders within the IHC 3 + population. While IHC 3 + tumors as a whole showed an ORR of 50%, mPFS of 4.9 months and mOS of 9.5 months, HER2-homogeneous cases achieved markedly superior outcomes (ORR 79%, mPFS 6.3 months, mOS 9.8 months). Conversely, the IHC 3 + heterogeneous subgroup showed poor outcomes (ORR 10%, DCR 60%, mPFS 3.1 months), highlighting the substantial outcome heterogeneity that exists within the conventionally defined IHC 3 + population. Importantly, even this subgroup achieved better response rates than salvage chemotherapy options such as FTD / TPI (ORR 4%, DCR 44%, mPFS 2.0 months) [47]. These findings suggest that IHC-based HER2 heterogeneity assessment may represent a practical and accessible approach for refining patient stratification for T-DXd, as it can be performed using routine pathological evaluation without additional specialized assays.

To explore potential therapeutic alternatives for HER2-heterogeneous tumors, we analyzed CLDN18.2 expression across our cohort. Although the median percentage of tumor cells with moderate-to-strong (≥ 2 +) CLDN18.2 membranous staining was numerically higher in HER2-heterogeneous tumors (65%) compared to HER2-homogeneous tumors (35%), this difference was not statistically significant (p = 0.16). CLDN18.2 positivity was observed in 40% of HER2-homogeneous cases and 50% of HER2-heterogeneous cases (p = 0.55). Notably, among HER2-heterogeneous patients half demonstrated CLDN18.2 positivity, suggesting potential eligibility for anti-CLDN18.2 therapy.

ILD is the most clinically significant AE associated with T-DXd. A higher trend of ILD in the homogeneity group (35% vs. 9%, p = 0.06) was observed in 9 (21%) patients in this study (Table 5). Previously reported ILD incidence in gastric cancer ranges from 9.1–14.9% [24, 30, 48, 49], and in breast cancer, although the background differs from 10.3–16.7% [50]. Risk factors include absence of primary tumor, low tumor burden [49], age < 65 years, Japanese ethnicity, presence of lung comorbidities, moderate/severe renal impairment, time since diagnosis > 4 years, T-DXd dose > 6.4 mg/kg, and oxygen saturation < 95% [51]. In our study, the slightly higher ILD incidence than previously reported may be attributed to the Japanese population, inclusion of potential ILD cases (both not confirmed by radiologists or pulmonologists and suspected), and the relatively longer treatment duration, particularly in the homogeneity group (Table S4). The association between the onset of ILD and longer duration or higher doses have been reported previously [52, 53]. Therefore, the higher frequency of ILD in the homogeneity group may be explained by longer treatment duration, higher dosage, and more frequent administration, as these factors were more common in the HER2-homogeneity group than in the HER2-heterogeneity group.

Several limitations should be acknowledged. The single-center retrospective design with a relatively small sample size may limit generalizability, and the cutoff value for HER2- heterogeneity was determined using time-dependent ROC analysis within the same dataset used for outcome evaluation. While this approach is acceptable for exploratory biomarker discovery, it introduces a potential risk of overfitting, and the observed differences may partly reflect this optimization process. External validation in an independent cohort will be essential to confirm the robustness and clinical utility of this biomarker. In addition, re-biopsy analysis was limited to eight patients, precluding definitive conclusions about dynamic HER2 changes. Moreover, HER2-positive proportion was evaluated by a single pathologist, and no molecular validation was performed. Future evaluation of gene amplification in ctDNA and re-biopsy specimens is desirable. These findings warrant validation in larger, multi-institutional cohorts. Additional analyses using continuous CLDN18.2-positive tumor cell percentage did not reveal a significant association with HER2 heterogeneity status (Figure S6b; Table S5), although sensitivity may be limited in this small cohort.

Nevertheless, our findings may have clinical implications. Identification of HER2- heterogeneity as a factor independently associated with T-DXd efficacy using pre-treatment specimens suggests that routine pathological assessment could include evaluation of HER2-positive proportions, not just binary HER2 status. The differential prognostic value of TFI based on HER2-heterogeneity represents a potential insight for refining patient stratification. Specifically, in patients with homogeneous HER2 expression, TFI may be considered as an additional prognostic factor when planning treatment sequences and discussing prognosis.

## Conclusion

Intratumoral HER2 heterogeneity in pre-treatment diagnostic specimens was associated with T-DXd efficacy and may represent a potential prognostic factor. Further prospective studies in independent, larger cohorts are warranted to validate these findings, to establish standardized methods for assessing HER2 heterogeneity and optimal cutoff values, and to explore potential molecular correlates.

## Supplementary Information

Below is the link to the electronic supplementary material.

Supplementary Material 1

Supplementary Material 1

Supplementary Material 1

Supplementary Material 1

Supplementary Material 1

Supplementary Material 1

Supplementary Material 1

Supplementary Material 1

Supplementary Material 1

Supplementary Material 1

Supplementary Material 1

## Data Availability

The data used in this study, although not publicly available due to privacy restrictions, will be made available to other researchers upon reasonable request.
