# Supplementary material for: Clinical impact of intratumoral HER2 heterogeneity on trastuzumab deruxtecan efficacy in patients with HER2-positive gastric cancer
Source: Gastric Cancer. 2026 Apr 2;29(3):597–610. doi: 10.1007/s10120-026-01736-9 (PMC13124857; doi:10.1007/s10120-026-01736-9)
Supplement: Supplementary file 1 — Supplementary Material 1 [file 10120_2026_1736_MOESM1_ESM.pptx]

## Slide 1
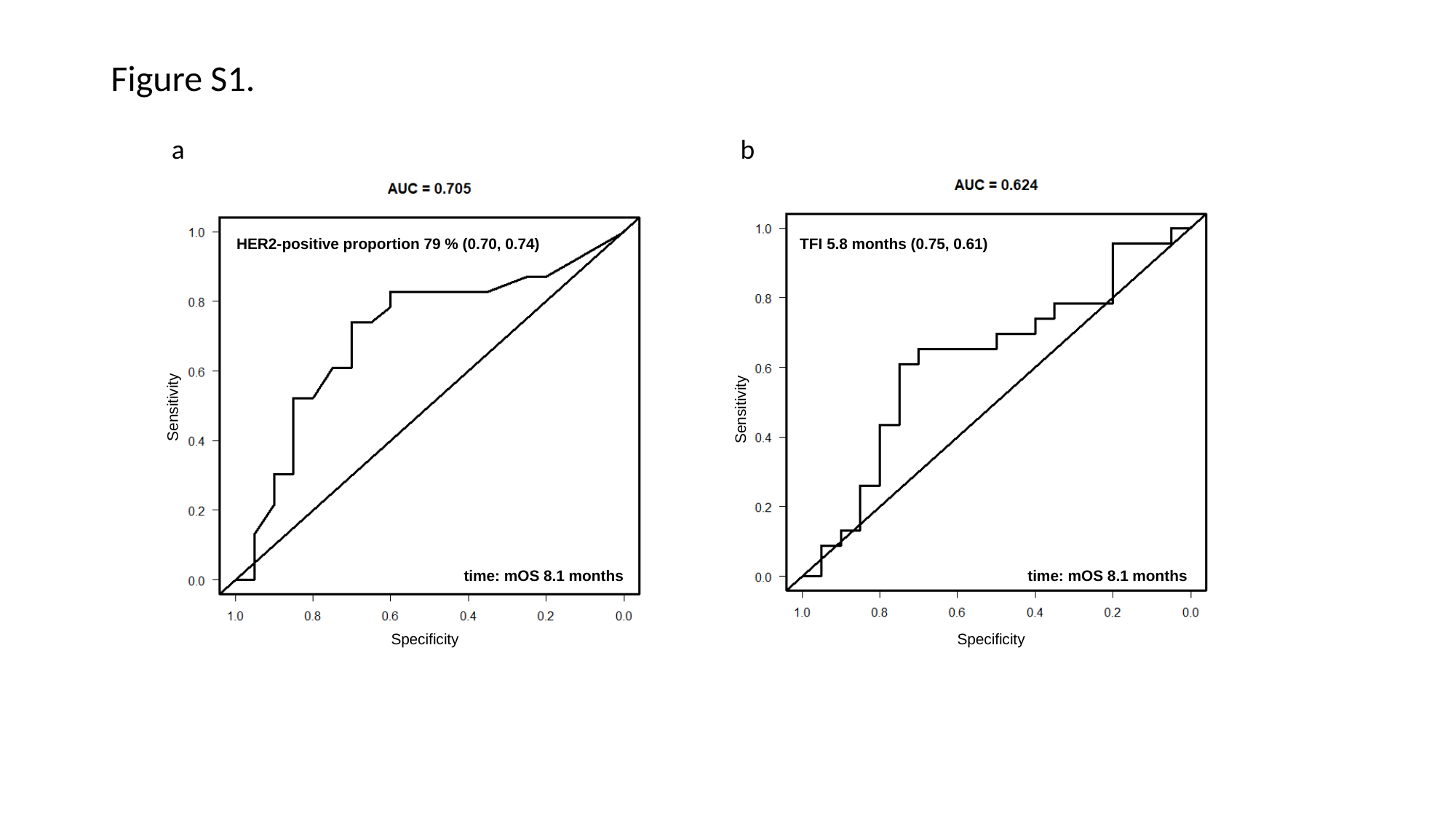

# Figure S1.
a
b
HER2-positive proportion 79 % (0.70, 0.74)
TFI 5.8 months (0.75, 0.61)
Sensitivity
Sensitivity
time: mOS 8.1 months
time: mOS 8.1 months
Specificity
Specificity
