# Supplementary material for: Clinical impact of intratumoral HER2 heterogeneity on trastuzumab deruxtecan efficacy in patients with HER2-positive gastric cancer
Source: Gastric Cancer. 2026 Apr 2;29(3):597–610. doi: 10.1007/s10120-026-01736-9 (PMC13124857; doi:10.1007/s10120-026-01736-9)
Supplement: Supplementary file 2 — Supplementary Material 1 [file 10120_2026_1736_MOESM2_ESM.pptx]

## Slide 1
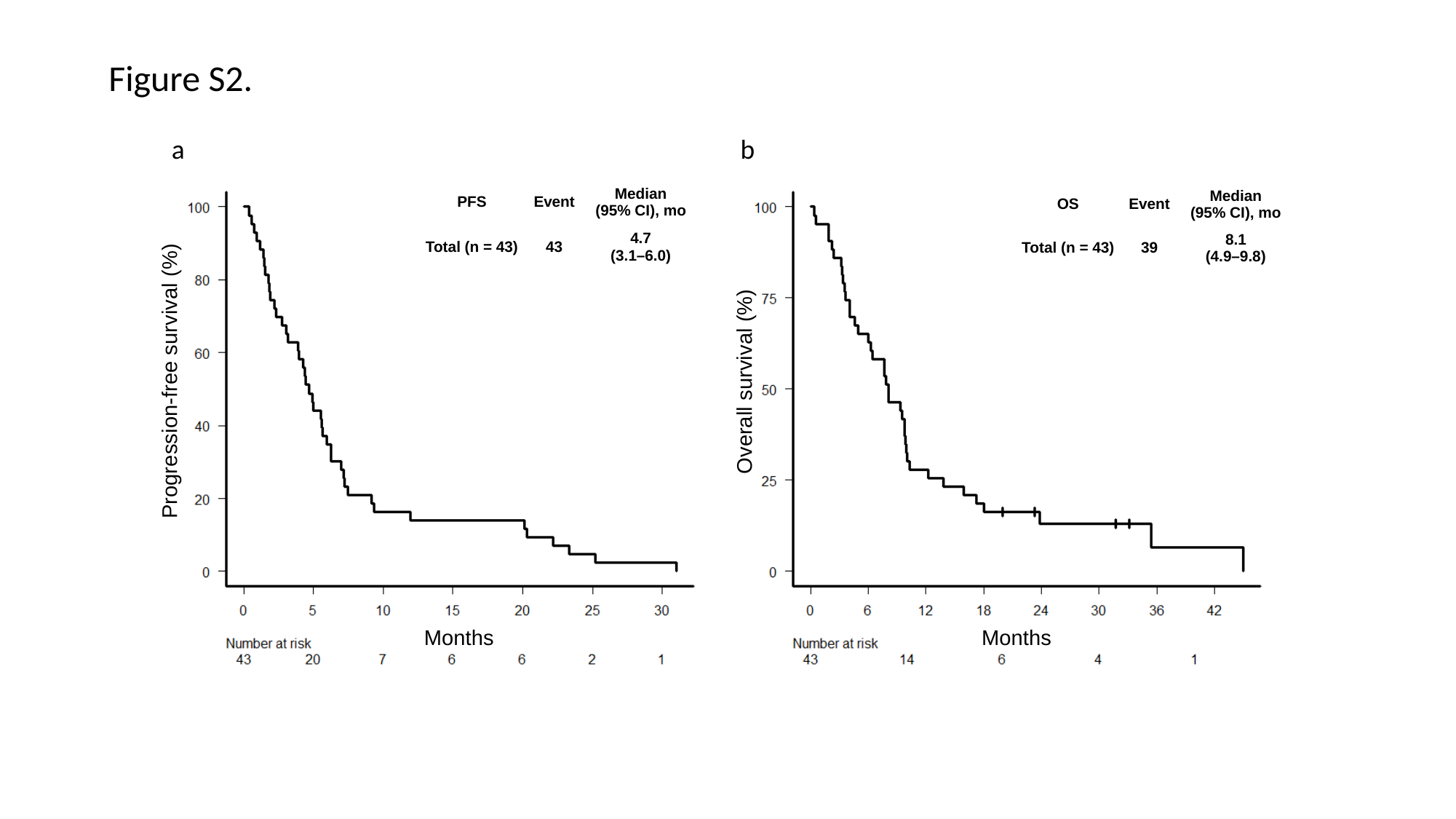

# Figure S2.
a
b
| PFS | Event | Median (95% CI), mo |
| --- | --- | --- |
| Total (n = 43) | 43 | 4.7 (3.1–6.0) |
| OS | Event | Median (95% CI), mo |
| --- | --- | --- |
| Total (n = 43) | 39 | 8.1 (4.9–9.8) |
Progression-free survival (%)
Overall survival (%)
Months
Months
