# Supplementary material for: Clinical impact of intratumoral HER2 heterogeneity on trastuzumab deruxtecan efficacy in patients with HER2-positive gastric cancer
Source: Gastric Cancer. 2026 Apr 2;29(3):597–610. doi: 10.1007/s10120-026-01736-9 (PMC13124857; doi:10.1007/s10120-026-01736-9)
Supplement: Supplementary file 3 — Supplementary Material 1 [file 10120_2026_1736_MOESM3_ESM.pptx]

## Slide 1
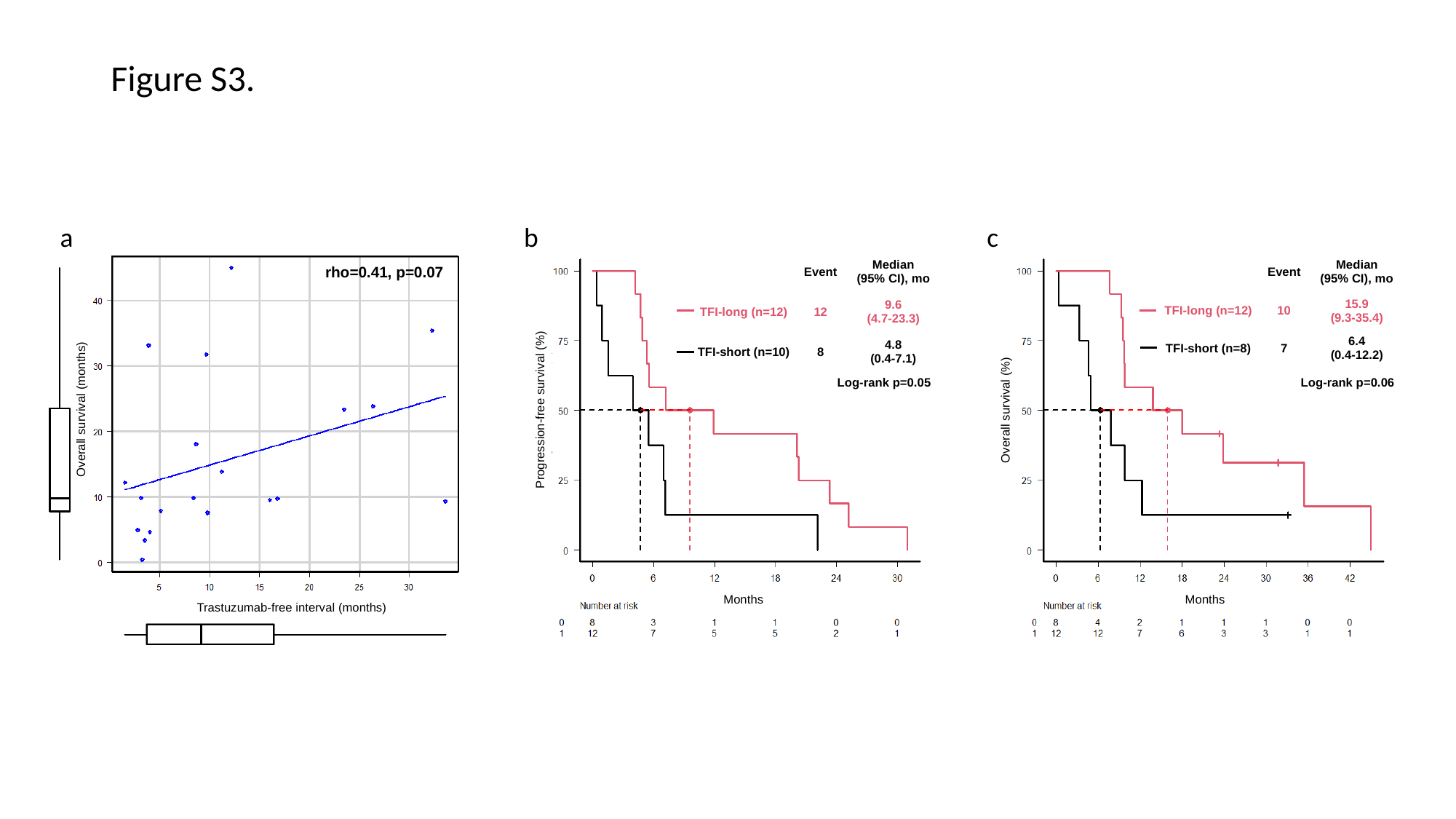

# Figure S3.
a
b
c
| | Event | Median (95% CI), mo |
| --- | --- | --- |
| TFI-long (n=12) | 12 | 9.6 (4.7-23.3) |
| TFI-short (n=10) | 8 | 4.8 (0.4-7.1) |
| | Event | Median (95% CI), mo |
| --- | --- | --- |
| TFI-long (n=12) | 10 | 15.9 (9.3-35.4) |
| TFI-short (n=8) | 7 | 6.4 (0.4-12.2) |
rho=0.41, p=0.07
Log-rank p=0.05
Log-rank p=0.06
Progression-free survival (%)
Overall survival (%)
Overall survival (months)
Months
Months
Trastuzumab-free interval (months)
