# Supplementary material for: Clinical impact of intratumoral HER2 heterogeneity on trastuzumab deruxtecan efficacy in patients with HER2-positive gastric cancer
Source: Gastric Cancer. 2026 Apr 2;29(3):597–610. doi: 10.1007/s10120-026-01736-9 (PMC13124857; doi:10.1007/s10120-026-01736-9)
Supplement: Supplementary file 4 — Supplementary Material 1 [file 10120_2026_1736_MOESM4_ESM.pptx]

## Slide 1
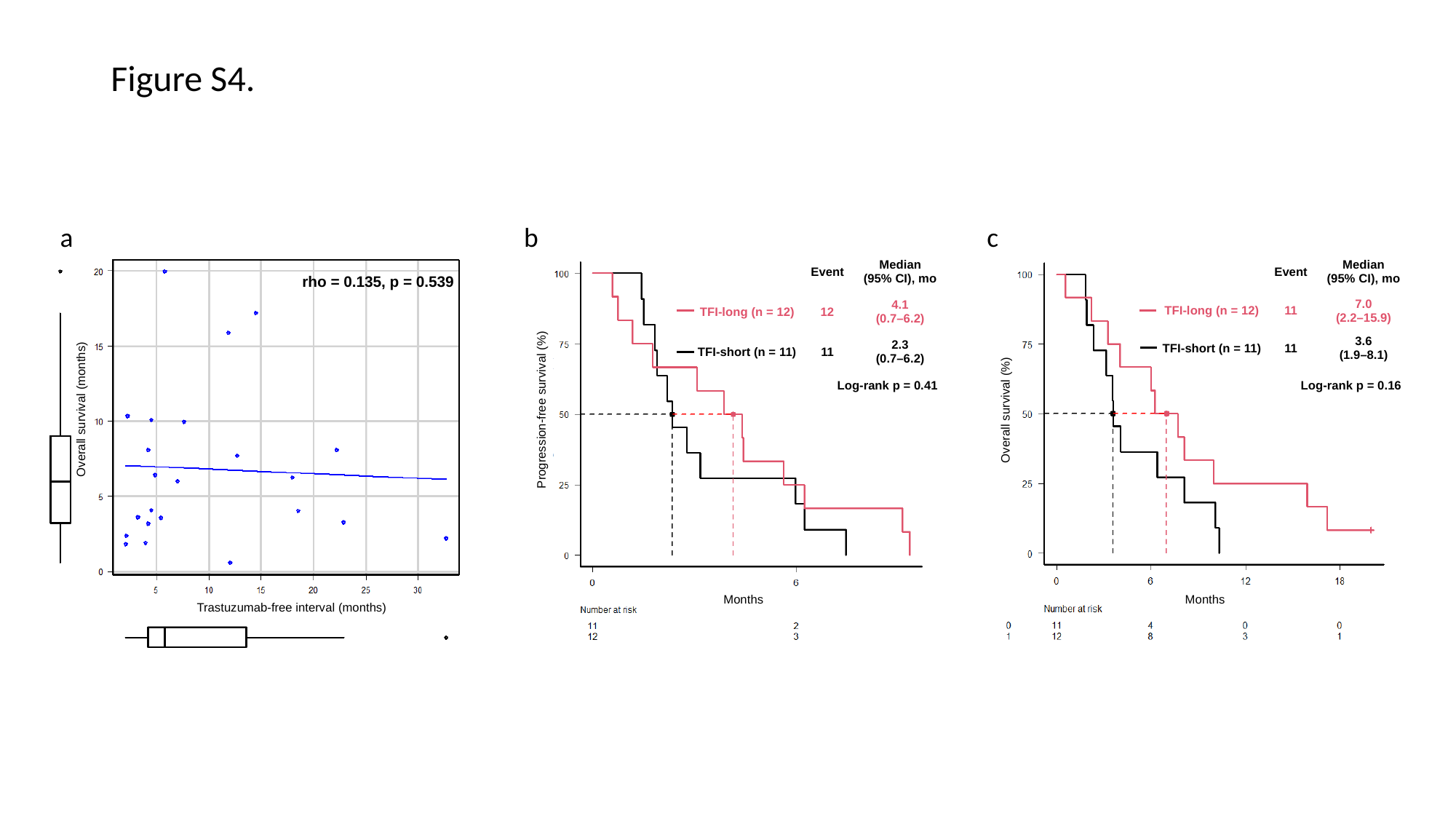

# Figure S4.
a
b
c
| | Event | Median (95% CI), mo |
| --- | --- | --- |
| TFI-long (n = 12) | 12 | 4.1 (0.7–6.2) |
| TFI-short (n = 11) | 11 | 2.3 (0.7–6.2) |
| | Event | Median (95% CI), mo |
| --- | --- | --- |
| TFI-long (n = 12) | 11 | 7.0 (2.2–15.9) |
| TFI-short (n = 11) | 11 | 3.6 (1.9–8.1) |
rho = 0.135, p = 0.539
Log-rank p = 0.41
Log-rank p = 0.16
Progression-free survival (%)
Overall survival (%)
Overall survival (months)
Months
Months
Trastuzumab-free interval (months)
