# Supplementary material for: Clinical impact of intratumoral HER2 heterogeneity on trastuzumab deruxtecan efficacy in patients with HER2-positive gastric cancer
Source: Gastric Cancer. 2026 Apr 2;29(3):597–610. doi: 10.1007/s10120-026-01736-9 (PMC13124857; doi:10.1007/s10120-026-01736-9)
Supplement: Supplementary file 5 — Supplementary Material 1 [file 10120_2026_1736_MOESM5_ESM.pptx]

## Slide 1
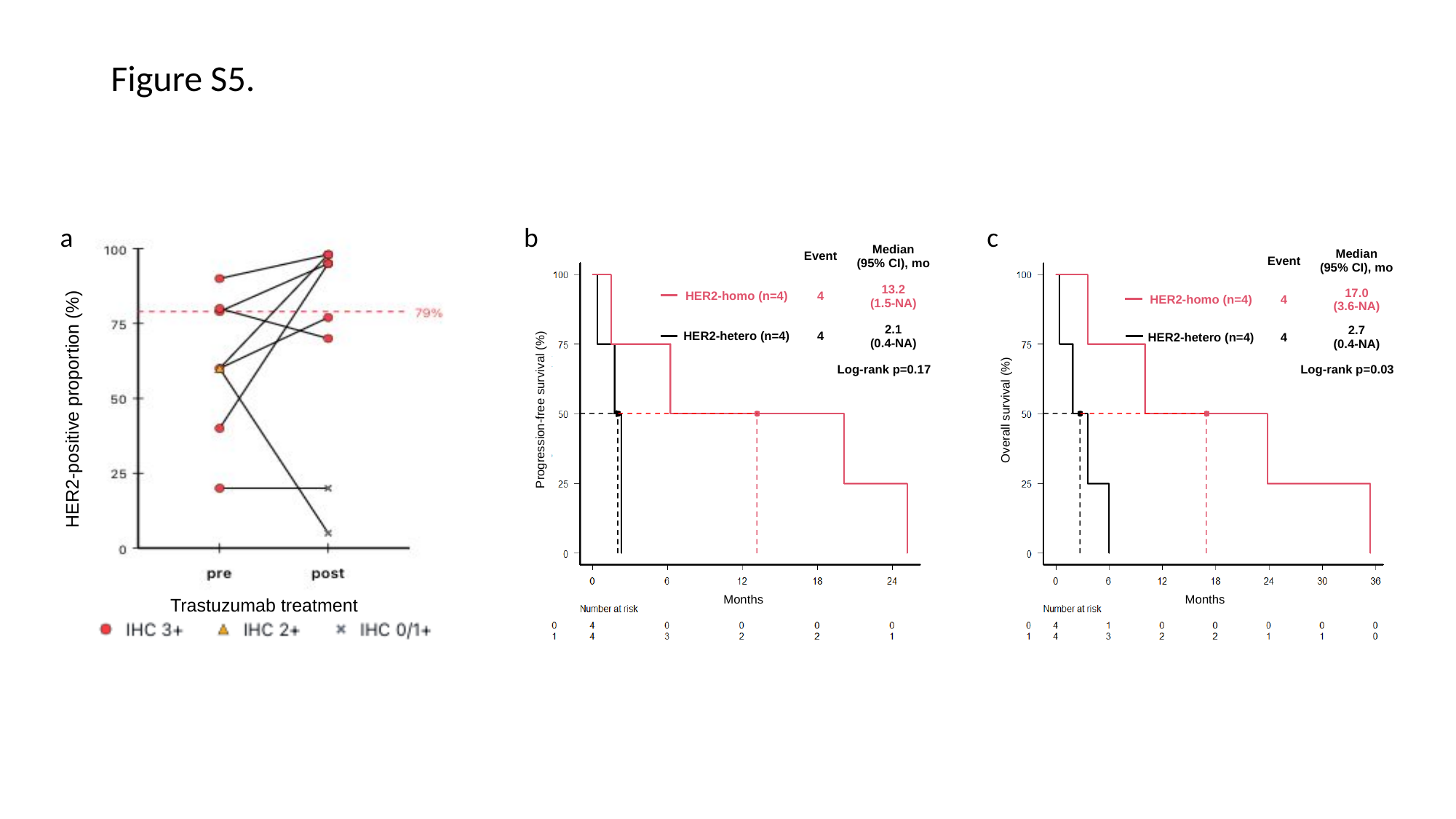

# Figure S5.
a
b
c
| | Event | Median (95% CI), mo |
| --- | --- | --- |
| HER2-homo (n=4) | 4 | 13.2 (1.5-NA) |
| HER2-hetero (n=4) | 4 | 2.1 (0.4-NA) |
| | Event | Median (95% CI), mo |
| --- | --- | --- |
| HER2-homo (n=4) | 4 | 17.0 (3.6-NA) |
| HER2-hetero (n=4) | 4 | 2.7 (0.4-NA) |
Log-rank p=0.17
Log-rank p=0.03
HER2-positive proportion (%)
Progression-free survival (%)
Overall survival (%)
Months
Months
Trastuzumab treatment
