# Supplementary material for: Clinical impact of intratumoral HER2 heterogeneity on trastuzumab deruxtecan efficacy in patients with HER2-positive gastric cancer
Source: Gastric Cancer. 2026 Apr 2;29(3):597–610. doi: 10.1007/s10120-026-01736-9 (PMC13124857; doi:10.1007/s10120-026-01736-9)
Supplement: Supplementary file 6 — Supplementary Material 1 [file 10120_2026_1736_MOESM6_ESM.pptx]

## Slide 1
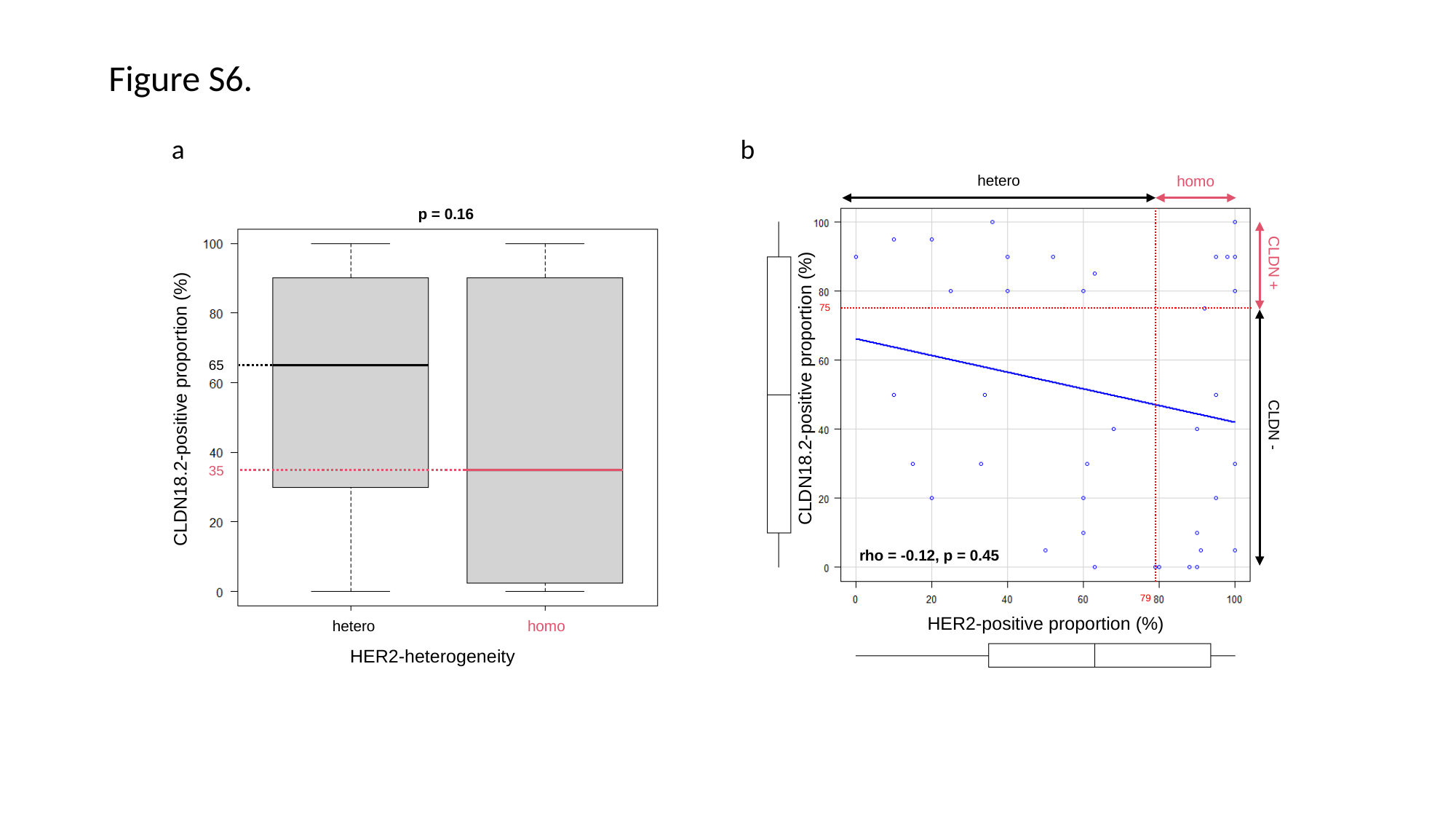

# Figure S6.
a
b
hetero
homo
p = 0.16
CLDN +
75
65
CLDN18.2-positive proportion (%)
CLDN18.2-positive proportion (%)
CLDN -
35
rho = -0.12, p = 0.45
79
HER2-positive proportion (%)
homo
hetero
HER2-heterogeneity
